# The Auricles of the Heart Act by Their Elasticity and Contractility, Not by Muscles

**Published:** 1860-07

**Authors:** Charles Smith

**Affiliations:** New Orleans


### THE AURICLES OF THE HEART

### ACT BY THEIR ELASTICITY AND CONTRACTILITY, NOT BY MUSCLES.

BY CHARLES SMITH, M. D., NEW ORLEANS.

To demonstrate this fact, we shall first expose the heart, and then follow the current of the blood.

Tie the pulmonary veins above the auricle; perforate the mitral valves of the ventricle, and inject through the aorta, and fill the left ventricle and auricle to their fullest capacity, and lay the preparation aside until perfectly dry; when the auricle will appear as transparent as glass, and the ventricle perfectly opaque.

This proof that the auricles have no muscles, or muscular fibres, ought to convince any one who has not committed himself upon the subject. I must confess that I have often admitted to my professor that I could see the muscular fibres in the auricles; nor could I contradict it, until I had lectured upon anatomy and physiology myself, and given the subject special attention.

We say, then, that the auricles act, upon the principle of *elasticity* and *contractility*, dependent upon the ventricles. During the action of contraction of the ventricles, the auricles are distended with blood, and continue so until the re-action of the ventricle, when the blood flows (upon the principle of the laws of fluids) into the ventricle, which again contracts, and propels it into the arteries.

We may simply say here, that, if muscular action were necessary for the purpose of emptying the auricle, the pulmonary veins would have valves, to prevent the regurgitation of blood. But, as yet, none have ever been discovered. In all the course of the circulation, we find valves in proportion to the force applied. Hence we might reasonably infer that the auricles do not really act—only passively.

The idea, then, that muscular fibres could be seen in the auricles, I believe to be an error that ought to be corrected; and if they can be shown to exist, then it is certain that the circulation of the blood does not obey the laws of force, and motion, and fluids.

If this view, then, be correct, the anricle is a passive, not an active appendage, and the blood would be acted upon the same as it would in the suction pump, where the column of water is subservient to the action of the piston.

So, in the circulation, the blood in the auricles depends upon the action of the ventricles. If passive, the auricles are only *reservoirs*, and adapt themselves to the amount of blood required for the use of the ventricles.

In the structure of the heart, we see the vast difference between the right and left ventricles, in the compartive thickness of their parietes and the remarkably great strength of valves, to prevent reflux—all adapted to the two circulations, the general system and the pulmonary.

Now, if it is necessary to provide against regurgitation in one part of the circulation, where active force is used, it must be in all; therefore, if there were any muscular action, or other kind but passive, there would cetainly be valves at the auricles, or in the course of the pulmonary veins, otherwise the capillary circulation would be completely arrested, and the grandest object in the circulation defeated.—*N. O. Med. and Surg. Journal.*

